# Integrative Approach to *Phlebotomus mascittii* Grassi, 1908: First Record in Vienna with New Morphological and Molecular Insights

**DOI:** 10.3390/pathogens9121032

**Published:** 2020-12-09

**Authors:** Edwin Kniha, Vít Dvořák, Petr Halada, Markus Milchram, Adelheid G. Obwaller, Katrin Kuhls, Susanne Schlegel, Martina Köhsler, Wolfgang Poeppl, Karin Bakran-Lebl, Hans-Peter Fuehrer, Věra Volfová, Gerhard Mooseder, Vladimir Ivovic, Petr Volf, Julia Walochnik

**Affiliations:** 1 Institute of Specific Prophylaxis and Tropical Medicine, Center for Pathophysiology, Infectiology and Immunology, Medical University of Vienna, 1090 Vienna, Austria; edwin.kniha@meduniwien.ac.at (E.K.); martina.koehsler@meduniwien.ac.at (M.K.); 2 Department of Parasitology, Faculty of Science, Charles University Prague, 128 43 Prague, Czech Republic; vidvorak@natur.cuni.cz (V.D.); veravolf@seznam.cz (V.V.); volf@cesnet.cz (P.V.); 3 BioCeV, Institute of Microbiology of the Czech Academy of Sciences, 252 50 Vestec, Czech Republic; halada@biomed.cas.cz; 4 Department of Integrative Biology and Biodiversity Research, Institute of Zoology, University of Natural Resources and Life Sciences Vienna, 1180 Vienna, Austria; markusmilchram@gmx.net; 5 Federal Ministry of Defence, Division of Science, Research and Development, 1090 Vienna, Austria; adelheid.obwaller@bmlv.gv.at; 6 Division Molecular Biotechnology and Functional Genomics, Technical University of Applied Sciences Wildau, 15745 Wildau, Germany; katrin.kuhls@th-wildau.de (K.K.); susanne.schlegel@th-wildau.de (S.S.); 7 Research Platform “Models & Simulation”, Leibniz Centre for Agricultural Landscape Research (ZALF), 15374 Müncheberg, Germany; 8 Division Microsystems Engineering, Technical University of Applied Sciences Wildau, 15745 Wildau, Germany; 9 Department of Dermatology and Tropical Medicine, Military Medical Cluster East, Austrian Armed Forces, 1210 Vienna, Austria; wolfgang.poeppl@bmlv.gv.at (W.P.); gerhard.mooseder@bmlv.gv.at (G.M.); 10 Department of Pathobiology, Institute of Parasitology, University of Veterinary Medicine Vienna, 1210 Vienna, Austria; karin.bakran-lebl@vetmeduni.ac.at (K.B.-L.); hans-peter.fuehrer@vetmeduni.ac.at (H.-P.F.); 11 Department of Biodiversity, FAMNIT, University of Primorska, 6000 Koper-Capodistria, Slovenia; vladimir.ivovic@famnit.upr.si

**Keywords:** *Transphlebotomus*, Central Europe, autoimmunofluorescence, MALDI-TOF mass spectrometry, genotyping, leishmaniasis

## Abstract

Sand flies (Diptera: Psychodidae: Phlebotominae) are blood-feeding insects that transmit the protozoan parasites *Leishmania* spp. and various arthropod-borne (arbo) viruses. While in Mediterranean parts of Europe the sand fly fauna is diverse, in Central European countries including Austria mainly *Phlebotomus mascittii* is found, an assumed but unproven vector of *Leishmania infantum*. To update the currently understudied sand fly distribution in Austria, a sand fly survey was performed and other entomological catches were screened for sand flies. Seven new trapping locations of *Ph. mascittii* are reported including the first record in Vienna, representing also one of the first findings of this species in a city. Morphological identification, supported by fluorescence microscopy, was confirmed by two molecular approaches, including sequencing and matrix-assisted laser desorption/ionization-time of flight mass spectrometry (MALDI-TOF MS) protein profiling. Sand fly occurrence and activity were evaluated based on surveyed locations, habitat requirements and climatic parameters. Moreover, a first comparison of European *Ph. mascittii* populations was made by two marker genes, cytochrome c oxidase subunit 1 (*COI*), and cytochrome b (*cytb*), as well as MALDI-TOF mass spectra. Our study provides new important records of *Ph. mascittii* in Austria and valuable data for prospective entomological surveys. MALDI-TOF MS protein profiling was shown to be a reliable tool for differentiation between sand fly species. Rising temperatures and globalization demand for regular entomological surveys to monitor changes in species distribution and composition. This is also important with respect to the possible vector competence of *Ph. mascittii*.

## 1. Introduction

Phlebotomine sand flies (Diptera: Psychodidae: Phlebotominae) occur in tropical, subtropical, as well as temperate regions. They are of significant medical importance as vectors of *Leishmania* spp., bacteria and several arthropod-borne (arbo) viruses in various regions of both Old and New World. Leishmaniasis is among the most prominent and yet neglected infectious diseases [1,2].

In Europe, sand flies are endemic throughout most of the Mediterranean countries while their occurrence north of the Alps and in Central Europe was overlooked for a long time. *Phlebotomus mascittii* Grassi, 1908, and *Phlebotomus perniciosus* Newstead, 1911, were recorded in Germany for the first time in 1999 and 2001, respectively [3,4]. A decade later, *Ph. mascittii* was recorded in Austria and its presence further confirmed by several entomological surveys [5,6,7], while a single *Ph. mascittii* specimen was also collected in Slovakia close to the Austrian border [8]. Very recently, *Phlebotomus simici* Nitzulescu, 1931, was recorded for the first time in Austria [9]. Apart from these findings, knowledge on sand fly distribution, species diversity and ecological factors determining their occurrence in Central Europe is scarce.

*Ph. mascittii* is the most widely distributed species in Europe and known to occur in Spain, France including Corsica, Italy, Switzerland, Germany, Belgium, Austria, Slovakia, Slovenia, Croatia, Hungary, and Serbia [3,6,8,10,11,12,13,14,15,16,17,18,19], as well as Algeria in North Africa [20]. It is also the sand fly species with the northernmost distribution in the Palearctic, occurring in Germany as far as 50° North [11,15,21].

*Ph. mascittii* is a suspected vector for *Leishmania infantum* [22,23], however, its vector competence has not yet been experimentally proven, albeit repeated reports of autochthonous leishmaniasis cases in Germany and Austria may indicate possible involvement of *Ph. mascittii* in transmission of *Leishmania infantum* [24,25,26,27].

To update the hitherto underreported sand fly distribution in Austria, dedicated entomological field studies were performed and bycatches from other entomological surveys were investigated. Species identification of obtained specimens was achieved by both, morphology and two different molecular approaches, including DNA sequencing of the two mitochondrial genes *COI* and *cytb*, and matrix-assisted laser desorption/ionization-time of flight mass spectrometry (MALDI-TOF MS) protein profiling. Ecological and climatic parameters of *Ph. mascittii* records were evaluated to promote prospective trapping success.

## 2. Results

### 2.1. Sand Fly Trapping and Identification

**Figure 1:**
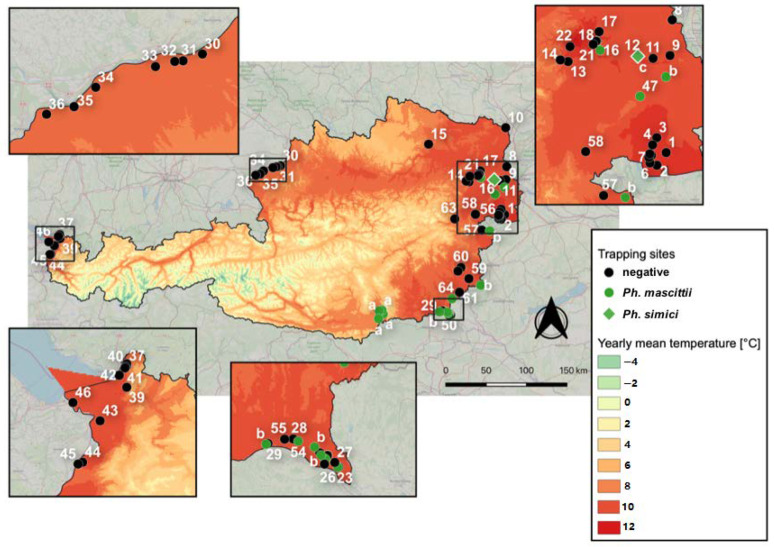
Distribution map of all published *Ph. mascittii* and *Ph. simici* records in Austria including all surveyed locations presented in this study. The climate map of Austria shows mean annual temperatures (1971–2000). Locations sampled within this study are marked with digits (Table S1), prior published records are marked with letters, (**a**) Naucke et al. 2011 [5], (**b**) Poeppl et al. 2013 [6], (c) Kniha et al. 2020 [9].

Altogether, 65 sampling sites in six federal states of Austria, namely Vienna, Lower Austria, Burgenland, Styria, Upper Austria, and Vorarlberg were surveyed (Table S1). At seven (10.8%) of these locations altogether 28 sand flies were trapped (Figure 1). Two (7.1%) were male and 26 (92.9%) were female, of which one specimen was engorged and three were gravid. The earliest capture was observed on 5 July and the latest capture was observed on 24 August.

In survey 1, two of the 46 (4.3%) sampled locations were positive. A single specimen was trapped in the 11th district of Vienna, the capital of Austria, on 17 July 2019 at a horse farm outside a barn used for hay storage. A second location was found positive in Laafeld, Styria, where one and two specimens were trapped in front of a chicken shed on 6 August 2018 and 5 August 2019, respectively. (Table 1). All locations surveyed in Upper Austria and Vorarlberg were negative (Figure 1).

In survey 2, only one location, namely Kaisersteinbruch in Lower Austria was sampled, where two specimens were caught on 11 and 26 July 2013 outside of a dog kennel (Table 1).

In survey 3, three of nine (33.3%) sampled locations were positive, which were all located in the federal district of Styria. In Hummersdorf, six and eight specimens were caught on 5 and 6 July 2015, respectively, inside and outside a chicken barn. In Bad Radkersburg, two specimens were caught on 6 July 2015 inside an old chicken shed and in Unterpurkla, four specimens were caught on 6 July 2015 inside a barn next to a chicken shed (Table 1).

In survey 4, screening bycatches of a mosquito monitoring, one of nine (11.1%) sampled locations were positive. Two sand fly specimens were trapped on 29 July and 24 August 2019 with a BG sentinel trap baited with CO_2_ in Neuhaus in the federal state of Burgenland (Table 1). The trap was set in the garden of a private property without animal barns or sheds. No animals were reported to live on the property and only rodents were observed to be present under a pile of wood close to the trap by the owners.

The majority of trapping sites, including those without sand fly catches, were animal farms with various domestic and farm animals as well as rodents present, being typical and suitable sand fly trapping sites.

Of all 28 caught specimens, 22 (78.6%) were caught with standard CDC light traps, four (14.3%) with standard CDC light traps with additional dry ice and two (7.1%) were caught as bycatch with BG sentinel traps using CO_2_ as bait. However, due to the low number of caught specimens and the fact that not all sampling techniques were applied at all locations, statistical analysis on trapping success by type of trap was not possible.

**Figure 2:**
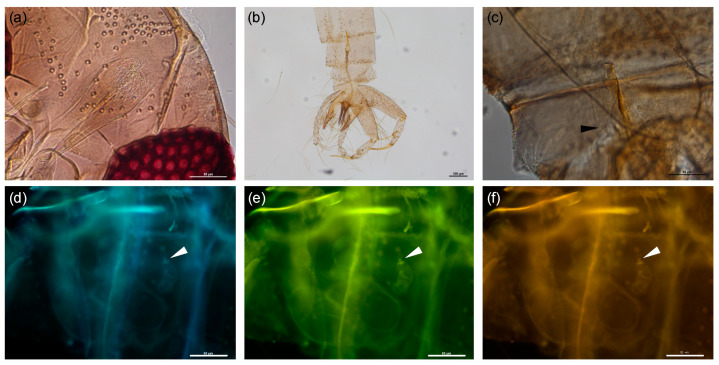
*Phlebotomus mascittii* characters for morphological identification. Pharynx with large irregular teeth (**a**), aedeagus with distal cup-like expansion (**b**), spermatheca not ringed, without neck indicated by black arrow (**c**), spermatheca under 330–380 nm light (**d**), spermatheca under 420–490 nm light (**e**), spermatheca under 585 nm light (**f**), tip of spermatheca with missing head is indicated by white arrow (**d**–**f**).

All specimens were morphologically identified as *Phlebotomus mascittii*. Further confirmation was achieved by fluorescence microscopy, all three applied light spectra providing detailed images of the spermathecae (Figure 2). Correct morphological identification as *Ph. mascittii* was confirmed by DNA sequencing of all specimens. Obtained sequences were compared to reference sequences stored in GenBank. Sequence similarities with sequences in GenBank ranged from 99.84% to 100% (KY848831.1, KX981913.1) for cytochrome c oxidase subunit 1 (*COI*) and from 98.44% to 100% (MG800324.1, KR336656.1) for cytochrome b (*cytb*).

**Figure 3:**
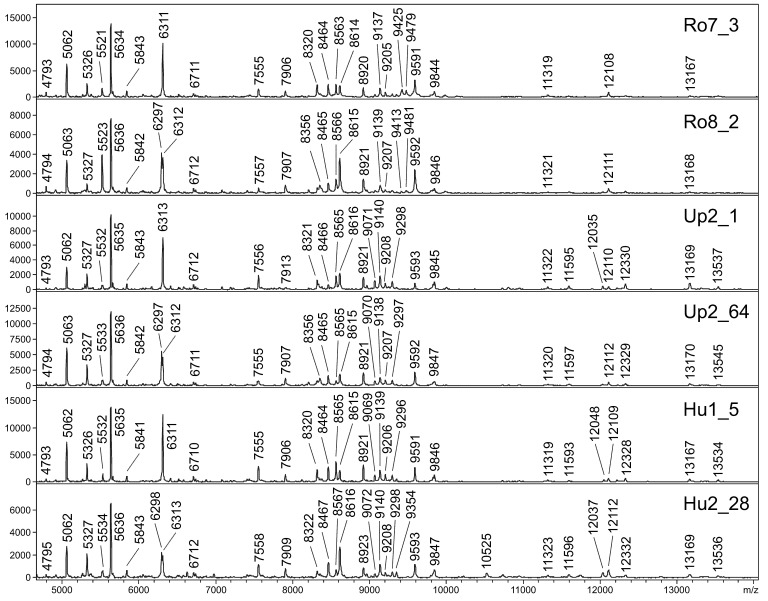
Matrix-assisted laser desorption/ionization-time of flight (MALDI-TOF) mass spectra of *Ph. mascittii* specimens originating from three different Austrian localities (Ro = Rohrau, Up = Unterpurkla, Hu = Hummersdorf).

**Figure 4:**
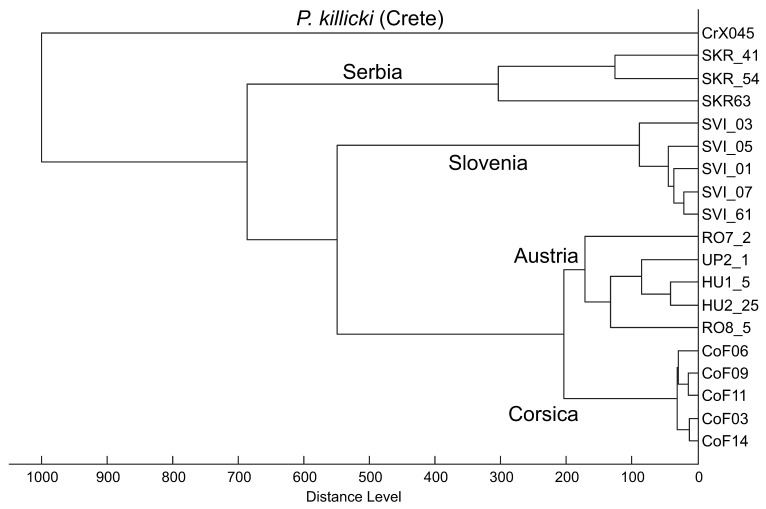
Dendrogram generated by cluster analysis of MALDI-TOF MS protein profiles of four geographically distant European populations of *Ph. mascittii*. As an outgroup, a mass spectrum of *Phlebotomus killicki* collected in Crete was used.

MALDI-TOF MS protein profiling provided reproducible mass spectra with a high number of intense signals for all analyzed specimens (Figure 3), males and females producing similar protein profiles. All 5 spectra of specimens from Austria confirmed the species identification of *Ph. mascittii*. When these protein profiles were compared with those of *Ph. mascittii* originating from three European countries (France, Slovenia, and Serbia), the dendrogram generated by cluster analysis provided clear clustering according to the geographical origin with distinct clusters for all four countries. The protein profile of *Ph. killicki*, a closely related species of the subgenus *Transphlebotomus*, formed a sister cluster to all analyzed *Ph. mascittii* specimens (Figure 4).

*Leishmania* spp. DNA was not detected by PCR in any of the female specimens.

### 2.2. Pairwise Distances of Transphlebotomus Species

Overall, 27 *COI* sequences of all five *Transphlebotomus* species with a final length of 566 bp were included in the comparative analyses (Table S3). Mean intraspecific distances ranged from 0.09% to 0.6%, the lowest being calculated for *Ph. mascittii* (Table 2). In general, mean interspecific distances ranged from 6.6% to 15.4% and from 10.7% to 15.4% between *Ph. mascittii* and other *Transphlebotomus* species (Table 2).

Altogether, 31 *cytb* sequences of all five *Transphlebotomus* species with a final length of 475 bp were included in the comparative analysis (Table S4). Mean intraspecific distances ranged from 0.2% to 1.9% (Table 2). Overall, mean interspecific distances ranged from 8.7% to 13.9% and from 12.9% to 14.7% between *Ph. mascittii* and other *Transphlebotomus* species (Table 2).

### 2.3. Haplotype Analysis of Ph. Mascittii from Austria and Other Countries

**Figure 5:**
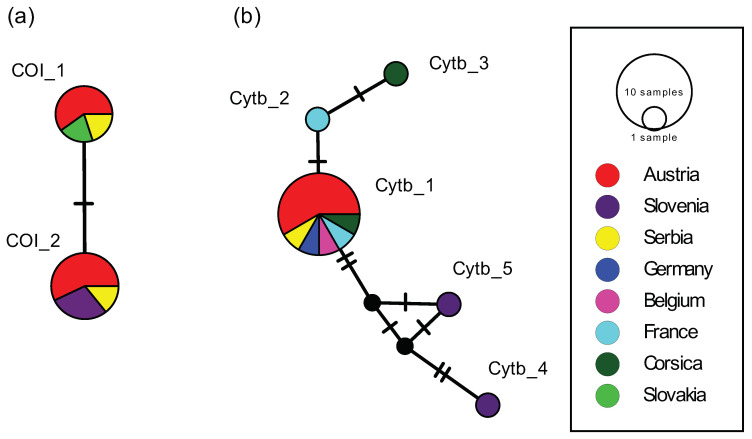
Haplotype networks of *Ph. mascittii* based on 12 *COI* sequences originating from 4 countries (**a**) and based on 16 *cytb* sequences originating from 6 countries (**b**).

Overall, 12 *COI* sequences with a final length of 613 bp were included in the analysis (Table S2) with an overall haplotype diversity (Hd) of 0.53 and an overall nucleotide diversity (π) of 0.53 (Table 3). The sequences of *Ph. mascittii*, originating from four different countries, namely Austria, Slovakia, Slovenia and Serbia, revealed two haplotypes (COI_1, COI_2) defined by one polymorphic site (Pos: 106, A/G) (Figure 5a). Interestingly, both haplotypes were found among the Austrian specimens. The specimens from the Austrian federal states Vienna and Styria shared one haplotype (COI_1) with specimens from Pernek, Slovakia, and Vojdovina, Serbia. The other Austrian specimens from Lower Austria, Burgenland, and Styria shared the other haplotype (COI_2) with specimens from Velike Zabjle and Cetore, Slovenia, as well as Krasava, Serbia (Table 3).

Altogether, 16 *cytb* sequences with a final length of 475 bp were used for the analysis (Table S3) with an overall haplotype diversity (Hd) of 0.45 and an overall nucleotide diversity (π) of 0.19 (Table 3). Sequences of *Ph. mascittii* originating from six different countries, namely France including Corsica, Belgium, Germany, Austria, Slovenia, and Serbia, revealed five haplotypes (Cytb_1–Cytb_5) defined by seven variable sites, of which three were parsimony informative (Figure 5b). In the *cytb* analysis, all Austrian specimens belonged to a single haplotype (Cytb_1), shared with specimens from Belgium, France including Corsica, Germany, and Serbia (Table 3).

### 2.4. Climatic Parameters

**Figure 6:**
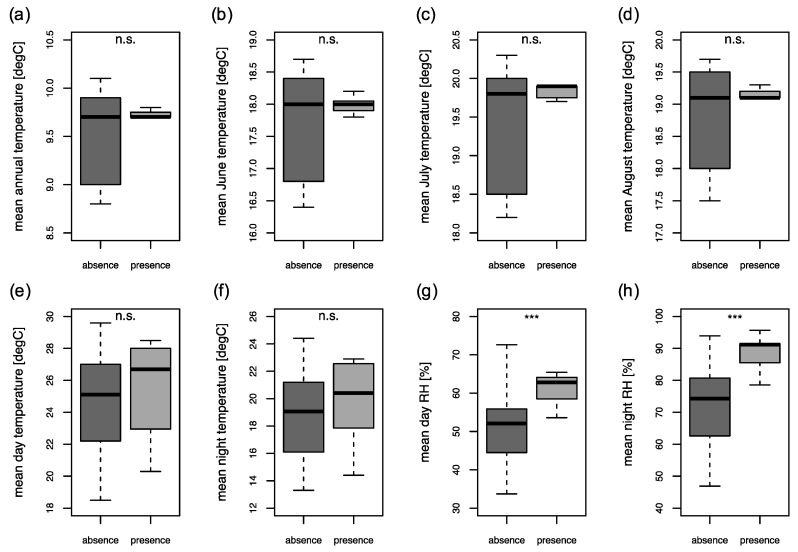
Comparison of climatic parameters at positive and negative surveyed locations analyzed by Kruskal–Wallis test. Mean annual temperature (**a**), mean June temperature (**b**), mean July temperature (**c**) and mean August temperature (**d**), parameters on temperature (**e**,**f**) and on relative humidity (RH) (**g**,**h**). Statistical differences between absence and presence are shown as not significant (n.s.), or as significant by asterisks (*** *p* < 0.001).

No significant differences were observed between annual mean temperature (Kruskal–Wallis test, *p* = 0.941), mean June temperature (Kruskal–Wallis test, *p* = 0.548), mean July temperature (Kruskal–Wallis test, *p* = 0.889) and mean August temperature (Kruskal–Wallis test, *p* = 0.883) at successful and unsuccessful trapping sites (Figure 6a–d).

Further, no significant differences were observed between mean day temperatures (Kruskal–Wallis test, *p* = 0.344) and mean night temperatures (Kruskal–Wallis test, *p* = 0.435) at successful and unsuccessful trapping sites (Figure 6e,f). Sand flies were found to be active between 14.1 and 22.9 °C (mean = 19.8, SD = 2.9) mean night temperature (Figure 6f). Minimum night temperatures during successful trap nights ranged from 13.5 to 21.4 °C.

However, significant differences based on successful and unsuccessful trap nights were observed between mean day relative humidity (RH) (Kruskal–Wallis test, *p* < 0.001) and mean night RH (Kruskal–Wallis test, *p* < 0.001) (Figure 6g,h). Sand flies were found to be active between 78.6% and 95.7% (mean = 88.0, SD = 7.8) mean night RH (Figure 6h).

## 3. Discussion

This study reports new findings of *Phlebotomus mascittii* in Austria, including the first record of this species in the capital Vienna, representing the northernmost record in Austria. In total, *Ph. mascittii* was found at seven new locations within four surveys, suggesting that its distribution in Austria may be wider than previously thought. We provide an integrative approach for its identification, using morphological as well as molecular methods.

*Ph. mascittii* is the type species of the subgenus *Transphlebotomus* Artemiev, 1984, which comprises four other species, namely *Phlebotomus canaaniticus* Adler and Theodor, 1931, *Phlebotomus economidesi* Léger, Depaquit, and Ferté, 2000, *Phlebotomus killicki* Dvořák, Votýpka and Volf, 2015, and *Phlebotomus anatolicus* Kasap, Depaquit, and Alten, 2015. A recent description of the latter two species from localities in the eastern Mediterranean region where composition of sand fly fauna had been repeatedly studied in the past underlines the challenges of species identification based on morphology within this subgenus [28]. Therefore, molecular approaches are useful to confirm species identification and the natrium dehydrogenase subunit 4 (*NADH4*) gene was presented as an informative marker for interspecific and intraspecific analysis within the *Transphlebotomus* subgenus [11].

Here, we applied three different techniques, including morphology, sequencing of the two commonly used mitochondrial marker genes *COI* and *cytb* and MALDI-TOF MS protein profiling. All specimens were morphologically identified to the species level, however, some individuals had hardly visible spermathecae, making morphological discrimination challenging. We further evaluated the usage of fluorescence microscopy to detect the autofluorescent spermathecae of *Ph. mascittii*, which we had already used for the identification of *Phlebotomus simici* [9]. By using different wavelengths of light the typical microstructures of the spermatheca of *Ph. mascittii* were visualized in high detail. Verification of morphological results was achieved with both mitochondrial marker genes used, *COI* and *cytb*. In general, interspecific distances exceeded 10% in both marker genes, with one exception, interspecific distances between *Ph. canaaniticus* and *Ph. anatolicus* were 6.6 and 8.7% for *COI* and *cytb*, respectively. This is in concordance with Kasap et al. [28], who observed similar interspecific distances with both marker genes and identified these two species as sister species.

MALDI-TOF MS protein profiling also confirmed species identification of Austrian specimens, providing distinct, species-specific protein profiles. This mass spectrometry method represents a currently emerging tool for species identification of various organisms including medically important arthropods that becomes favorable as a cost- and labor-effective alternative approach especially in field studies. However, in sand flies, it has been mostly used in studies focusing on the species diversity in rather small areas, often in foci of human leishmaniasis [29,30]. We attempted for the first time to compare protein profiles of distant populations of one species on a much larger geographical scale, analyzing specimens of *Ph. mascittii* from Austria, the French island of Corsica, Slovenia and Serbia. Our results clearly show that this method differentiates between these geographically distinct populations while successfully identifying all as *Ph. mascittii* when compared to the closely related species *Ph. killicki*. Specimens from a single locality in Corsica form a very compact cluster while specimens from Austria and Slovenia that originate from several localities show minor differences and form longer branches within their clusters. Specimens from Serbia were all collected at one locality, yet they also form longer branches, presumably due to the lower quality of obtained spectra because of compromised storage conditions.

Altogether, only seven out of the 64 surveyed locations were found positive for *Ph. mascittii*, even though most of the sampled locations were considered ideal for sand fly occurrence regarding annual mean temperature and potential breeding sites. Typically, only small trapping numbers were observed, as has been commonly reported from previous entomological surveys on sand flies in Austria [5,6] and bordering countries [8,31]. The attractiveness of light traps to *Ph. mascittii* is still a matter of discussion. However, eight individuals were trapped by a standard CDC light trap during a single night in Hummersdorf, indicating that light trapping is an appropriate technique for *Ph. mascittii*. In addition to this trapping method, trappings were also successful using CDC light traps with additional dry ice and BG sentinel traps baited with CO_2_ as an attractant. Successful trappings have also been reported with other methods, e.g., sticky traps [12,18,32], malaise traps [23] and emergence traps in a cellar in Switzerland [15], all nevertheless resulting in rather low trapping numbers. High numbers were reported from trappings with light traps in Sessa, southern Switzerland, where over 280 *Ph. mascittii* specimens were caught during a survey from 1987 to 1989 [15]. Comparative field studies are necessary to optimize trapping methods and attractants for *Ph. mascittii*. Based on our findings of *Ph. mascittii* in the frame of mosquito monitoring and the variety of suitable trapping methods for this species, the inspection of bycatch of entomological studies presents an opportunity to detect small overlooked *Ph. mascittii* populations.

The generally low population densities found might not only depend on the trapping method, but might be due to *Ph. mascittii* having specific ecological niches within typical habitats or might even depend on other yet unknown factors. As also observed in this study, *Ph. mascittii* in Central Europe is frequently found in old barns and sheds close to human dwellings and animals [3,5,6,8], even though *Ph. mascittii* has been proposed to be cavernicolous [11]. *Ph. mascittii* has been found to be active during winter time in a cave-like tunnel on the island of Corsica, where constant climatic conditions might increase population density and the time of activity [13]. A small number of individuals of this species have recently also been found in a small cave in Germany [31]. Despite these findings, records of such trapping sites are rather scarce. On the other hand, *Ph. mascittii* has been found to be present at other trapping sites such as wall crevices in Italy [12,32], dry stone walls at illegal waste sites in Slovenia [33] and in the basement of a house in Switzerland [15].

Despite the fact that global warming might promote the expansion of sand fly populations in Central Europe, the constant renovation and demolition of typical trapping sites such as old barns and sheds in Austria might be a potential limitation to the further dispersal, if other niches are not inhabited. This was observed while trapping in Upper Austria and Vorarlberg, where locating traditional old barns remained challenging, possibly one of the main reasons for not detecting sand flies in these areas so far, rather than climatic conditions.

In Vienna *Ph. mascittii* was found on a horse farm, of which there are many on the outskirts of Vienna but only very few towards the center. Even though climatic conditions are optimal in Vienna, typical breeding sites are scarce. Thus, alternative breeding sites should be evaluated and sampled within Vienna, to assess the potential spread of *Ph. mascittii* and the potential presence of other sand fly species within the city. The presence of sand flies in Vienna, with currently 1.9 million inhabitants, might be of public health relevance [22].

Temperature is one of the driving factors for sand fly occurrence and the 10 °C-annual-isotherm was proposed to be the boundary for northward dispersal of European sand fly species [34]. However, sand fly larvae overwinter in the ground and therefore we expect that temperatures during the summer season are more relevant for sand fly distribution. In this study, mean June, July, and August temperatures were slightly higher at successful trapping sites, but no significant differences compared to unsuccessful trapping sites were observed. Of all climatic parameters included in the analyses, only the relative humidity was significantly higher at successful sampling sites than at unsuccessful ones. Nonetheless, not all negative trapping sites can be explained by climatic conditions. *Phlebotomus mascitti* was observed to be active at night temperatures as low as 14.2 °C mean, which probably marks the lower boundary of sand fly activity and might affect population densities. The observed large activity spectrum between 14.2 °C and 22.9 °C is not surprising, as *Ph. mascittii* occurs in a variety of geographic regions including Central Europe, Southern Europe, and Northern Africa, where climatic conditions differ considerably. In addition, other factors such as relative humidity, precipitation and air pressure have an impact on sand fly activity during their active season and should be considered for field work [35,36].

Even though we did not detect *Leishmania* DNA in any of the sand fly females, such a monitoring is important. An anthropophilic behavior of *Ph. mascittii* was shown in Southern Switzerland [15] and the detection of *Leishmania infantum* DNA in an unfed female specimen in Austria [22] and on the Italian island of Montecristo [23] urge for further clarification of its suspected vector competence. Moreover, the frequent import of often asymptomatic *Leishmania*-infected dogs [37] adds another significant risk factor for the further spread of leishmaniasis.

The failure to trap *Ph. mascittii* in the federal states Vorarlberg and Upper Austria, of course, does not exclude that populations were overlooked. Further trappings over longer periods of time are necessary for confirmation. The absence of *Ph. mascittii* and other sand fly species in certain parts of Central Europe is most certainly a result of biogeographic events during and after the last glacial period [38]. During the last glacial period, Central Europe was most certainly free of sand flies and their return from Mediterranean refugial areas possibly started around 10,000 to 8000 years ago [39]. A maximum sand fly distribution in Central Europe was possibly reached during the Holocene optima around 6500 and 4500 years ago, respectively, and sand flies remained restricted to small favorable microclimatic habitats until today [34]. This hypothesis is supported by our haplotype networks which demonstrate close genetic relationships between European *Ph. mascittii* populations, indicating a rather recent dispersal. Although both marker genes, *COI* and *cytb*, are frequently used in phylogenetic studies [40], we could not detect substantial differences at the population level to further elucidate the origin of *Ph. mascittii* in Austria. Due to such very recent dispersal of *Ph. mascittii* within Europe, *COI* and *cytb* might not be ideal as marker genes and genes with higher mutation rates, such as the internal transcribed spacer 2, might be more suitable [41].

Overall, the Danube valley might constitute a corridor for sand fly dispersal, thereby connecting eastern and western populations. Whether eastern and western parts of Europe were post-glacially colonized from the same refugial area should be addressed in a detailed phylogeographic study, which would be crucial to understand the current sand fly distribution in Central Europe and to predict further sand fly dispersal in the future.

## 4. Material and Methods

### 4.1. Sand Fly Trapping and Available Material

The present study combines data of four different trapping surveys to update the current knowledge of sand fly distribution in Austria. Firstly, extensive field surveys to monitor sand flies were performed from 8 July to 21 August 2018 and 1 July to 25 August 2019, in six different federal states of Austria (survey 1). Trapping locations were chosen based on prevailing mean annual temperatures above 8 °C. In a second step, within these areas, suitable trapping sites were selected including farms, old barns, animal stables, private properties with and without animals present as well as two zoos. Every location was sampled for 2 to 3 consecutive nights based on weather conditions. Battery-operated CDC miniature light traps with fine gossamer collection bags (model #512, John W. Hock Company, Gainesville, FL, USA) were used. At outside locations, an additional source of CO_2_ was provided by a cooling bottle filled with ~700 g of dry ice/trap.

Secondly, material from prior sand fly trapping surveys in the years 2013 (survey 2) and 2015 (survey 3) with CDC light traps was included in this study.

Thirdly, bycatch of mosquito monitoring in the federal district Burgenland in July and August 2019 performed with BG sentinel traps baited with CO_2_ (Biogents AG, Regensburg, Germany) was screened for sand flies and included in this study (survey 4). Ethics Approval and Consent to Participate: Not applicable.

### 4.2. Climate Data

Mean annual, mean June, mean July and mean August temperatures of the trapping sites were accessed online (https://de.climate-data.org). Hourly temperature and relative humidity data of sampled locations were retrospectively obtained from the Central Institute for Meteorology and Geodynamics (ZAMG). Mean and minimum temperature and relative humidity were calculated for days (6 a.m. to 21 p.m.) and nights (22 p.m. to 5 a.m.).

### 4.3. Mapping of Sand Fly Distribution

Positive trapping sites as well as all previously published trapping locations in Austria were incorporated into a distribution map using Quantum GIS 3.4.11 [42].

### 4.4. Morphological Identification

Head and terminal segments of the abdomen of all caught sand fly specimens were dissected and slide-mounted in CMCP-10 mountant (Polysciences, Inc., Warrington, PA, USA). Identification was based on published morphological keys and descriptions of male genitalia, female spermatheca and pharyngeal armature [11,43]. Additionally, fluorescence microscopy was performed with a NIKON Eclipse E800 to identify hardly visible female spermatheca, as proposed in [9].

### 4.5. Molecular Identification by PCR and Sequencing

Molecular identification was performed by sequencing of the mitochondrial cytochrome c oxidase subunit 1 (*COI*) and cytochrome b (*cytb*) gene regions.

DNA was isolated from the remaining bodies with a QIAamp^®^ DNA Mini Kit 250 (QIAGEN, Hilden, Germany). PCR amplifications of the *COI* and *cytb* gene regions were performed with the primer pairs LCO-1490/CoxUniEr and CytbEf1/CytbEr2, respectively, as published in [9].

All PCR amplifications were performed with an Eppendorf Mastercycler (Eppendorf AG, Hamburg, Germany). The PCR products were subjected to electrophoresis in 2% agarose gels stained with GelRed^®^ Nucleic Acid Gel Stain (Biotium, Inc., Hayward, CA, USA). For further sequencing bands were analyzed with a Gel Doc^TM^ XR+ Imager (Bio-Rad Laboratories, Inc., Hercules, CA, USA), cut out from the gel and purified with the Illustra^TM^ GFX^TM^ PCR DNA and Gel Purification Kit (GE Healthcare, Buckinghamshire, UK). Sanger sequencing was performed with a Thermo Fisher Scientific SeqStudio (Thermo Fisher Scientific, Waltham, MA, USA). Sequences were obtained from both DNA strands and a consensus sequence was generated in GenDoc 2.7.0. Obtained sequences were compared to available sequences in the GenBank using the Basic Local Alignment Search Tool (BLAST) (https://blast.ncbi.nlm.nih.gov/Blast.cgi). One sequence of each location was submitted to GenBank: *COI* (MN812827.1–MN812830.1, MT332686.1–MT332688.1) and *cytb* (MN812832.1–MN812835.1, MT332689.1–MT332691.1).

To confirm the suitability of *COI* and *cytb* as marker genes to distinguish between *Transphlebotomus* species, mean interspecific and intraspecific Kimura-2-parameter (K2P) distances were calculated with MEGAX [44].

### 4.6. Identification by MALDI-TOF MS Protein Profiling

To confirm species identification of chosen specimens at surveyed localities (survey 3), MALDI-TOF MS protein profiling was applied according to previously optimized protocols for trapping and sample preparation [45,46]. Thoraxes of specimens trapped by CDC light traps were manually grinded by disposable pestles in 1.5 mL microtubes with 10 μL of 25% formic acid and briefly centrifuged at 10,000× *g*. Two μL of the homogenate were mixed with 2 μL of freshly prepared MALDI matrix, an aqueous 60% acetonitrile/0.3% TFA solution of sinapinic acid (30 mg/mL; Bruker Daltonics, Billerica, MA, USA). One μL of this mixture was applied on a steel MALDI plate in duplicates, air-dried and measured by Autoflex Speed MALDI-TOF spectrometer (Bruker Daltonics, Billerica, MA, USA) in a mass range of 4–25 kDa (20 × 300 laser shots from different positions of the sample spot). Obtained spectra were visualized by FlexAnalysis 3.4 software (Bruker Daltonics, Billerica, MA, USA), processed by MALDI Biotyper 3.1 and an in-house database that comprises reference spectra of 25 different sand fly species was searched to identify the species. Log score value (LSV) > 2.0 was decided as a threshold for an unambiguous identification. For MSP dendrogram creation, an individual main spectrum (MSP) was generated from each analyzed spectrum.

In total, 5 specimens from two Austrian localities (Hummersdorf and Unterpurkla) were subjected to MALDI-TOF MS protein profiling. For subsequent comparison of geographically distant European populations, we also included protein profiles of 4 specimens from another Austrian location (Rohrau) collected in 2018 [7] and three other European countries: 5 specimens (all males) collected in 2019 from a railway tunnel close to the road from Solenzara to Ste. Lucie de Porto Vecchio, Corsica, France [13], 3 specimens (1 male, 2 females) collected in 2016 from Krasava, Serbia [17] and 5 specimens (all females) collected in 2020 from Godovic, Ilirska Bistrica, Vrhnika, Zovnek and Velike Zablje, Slovenia. As an outgroup, a protein profile of *Phlebotomus killicki*, a closely related species of the subgenus *Transphlebotomus*, collected in 2019 in Xerokampos, Crete, Greece, was used [47] (Table S4).

### 4.7. Leishmania spp. Screening

Female specimens were screened by PCR for the presence of *Leishmania* parasites using the primers LITSR/L5.8S targeting the internal transcribed spacer 1 (ITS1) of the ribosomal DNA following the PCR protocol of protocol of El Tai et al. [48]. Five μL of extracted DNA from female sand flies was used in all PCR reactions. Microbial DNA free water (QIAGEN, Hilden, Germany) and 5 μL DNA of a male *Ph. mascittii* specimen were used as negative controls, 2 μL DNA of *Leishmania infantum* MHOM/TR/2000/OG-VL was used as a positive control.

### 4.8. Sequence Analyses

Obtained sequences were aligned with ClustalX 2.1 and edited with GeneDoc 2.7.0. for further analysis. DnaSP v.5 [49] was used to identify unique haplotypes. Median Joining Networks [50] were calculated and visualized with Popart v.1.7 [51].

### 4.9. Statistical Analyses

Data was analyzed with the R environment for Mac [52]. Descriptive statistics were performed with a one sample proportions test. A Shapiro–Wilk test was used to check for normality of the data. A nonparametric Kruskal–Wallis test was used to compare climate data. A two-sided *p*-value < 0.05 was considered statistically significant.

## Figures and Tables

**Table 1:** Sand fly positive locations surveyed in Austria.

Id	Location (Federal State)	Latitude, Longitude	Altitude (m.a.s.l.)	Trapping Site (Potential Host) ^a^	Specimen (Male/Female)	Species	Trap Date ^b^	Traps Set (Type)
47	Kaiser-steinbruch (Burgenland)	47.9911, 16.7103	179 m	outside, dog pound (dog)	1 (0m/1f)	*Ph. mascittii*	11.07.13	1 (CDC light)
					1 (0m/1f)	*Ph. mascittii*	26.07.13	1 (CDC light)
64	Neuhaus/Klausenbach (Burgenland)	46.8683, 16.0232	274 m	outside, human dwelling (rodents possibly)	1 (0m/1f)	*Ph. mascittii*	30.07.19	1 (BG sentinel & CO_2_)
					1 (0m/1f)	*Ph. mascittii*	25.08.19	1 (BG sentinel & CO_2_)
16	Neu Albern (Vienna)	48.1676, 16.4801	157 m	outside, roofed shed with hay (horse, dog)	1 (1m/0f)	*Ph. mascittii*	17.07.19	1 (CDC light & dry ice)
25	Laafeld (Styria)	46.6867, 16.0069	207 m	outside, chicken shed (chicken, geese, deer)	1 (0m/1f)	*Ph. mascittii*	06.08.18	1 (CDC light & dry ice)
					2 (0m/2f)	*Ph. mascittii*	05.08.19	1 (CDC light & dry ice)
48	Hummers-dorf (Styria)	46.7076, 15.9812	209 m	inside and outside, barn (chicken, dog)	6 (0m/6f)	*Ph. mascittii*	05.07.15	1 (CDC light)
					8 (1m/7f)	*Ph. mascittii*	06.07.15	2 (CDC light)
49	Bad Radkersburg (Styria)	46.7012, 15.9756	209 m	inside, old chicken shed (no)	2 (0m/2f)	*Ph. mascittii*	06.07.15	1 (CDC light)
54	Unterpurkla (Styria)	46.7319, 15.9062	223 m	inside, barn (chicken, cat)	4(0m/4f)	*Ph. mascittii*	06.07.15	1 (CDC light)

^a^ potential host present within a 50 m radius. ^b^ date of the evening when trap was set.

**Table 2:** Interspecific mean genetic distances (%) of the cytochrome c oxidase subunit 1 (*COI*) and cytochrome *b* (*cytb*) between the five *Transphlebotomus* species based on the Kimura-2-parameter model (*COI*/*cytb*). Diagonal bold values indicate intraspecific mean distances.

	Species	1	2	3	4	5
**1**	*Phlebotomus mascittii*	**0.09/0.3**				
**2**	*Phlebotomus canaaniticus*	10.7/14.7	**−/− ^a^**			
**3**	*Phlebotomus anatolicus*	10.9/13.9	6.6/8.7	**0.2/0.2**		
**4**	*Phlebotomus killicki*	12.2/12.9	11.4/13.7	12.1/12.7	**0.6/1.4**	
**5**	*Phlebotomus economidesi*	15.4/13.8	13.2/13.3	14.5/12.8	13.4/9.9	**− ^a^/1.9**

^a^ only one sequence available, included sequences are shown in Tables S2 and S3.

**Table 3:** Included *Ph. mascittii* specimens to the haplotype network analysis based on cytochrome c oxidase subunit 1 (*COI*) and cytochrome b (*cytb*) DNA sequences.

	*COI*		*cytb*		
Country, Location	GenBank	Haplotype	GenBank	Haplotype	Reference
France, Cévennes	-	-	KR336654.1	Cytb_2	Kasap et al. (2015)
Corsica, Porto Vecchio 1	-	-	KR336655.1	Cytb_3	Kasap et al. (2015)
Corsica, Porto Vecchio 2	-	-	KR336656.1	Cytb_1	Kasap et al. (2015)
Belgium, Saint-Cécile	-	-	KR336656.1	Cytb_1	Kasap et al. (2015)
Germany, Neuenburg	-	-	KR336656.1	Cytb_1	Kasap et al. (2015)
France, Haute-Pyrénées	-	-	HQ023281.1	Cytb_1	Mahamdallie et al. (2011)
Serbia, Vojdovina	KY848831.1	COI_1	-	-	Vaselek et al. (2017)
Serbia, Krasava	MN003381.1	COI_2	MK991774.1	Cytb_1	Vaselek et al. (2019)
Slovakia, Pernek	KX963380.1	COI_1	-	-	Dvořák et al. (2016)
Slovenia, Truske	-	-	MG800323.1	Cytb_4	Praprotnik et al. (2019)
Slovenia, Cetore	KX981916.1	COI_2	MG800324.1	Cytb_5	Hlavackova (GenBank)/Praprotnik et al. (2019)
Slovenia, Velike Zablje	KX869078.1	COI_2	-	-	Hlavackova (GenBank)
Austria, Burgenland, Kaisersteinbruch	MN812827.1	COI_2	MN812832.1	Cytb_1	present study
Austria, Burgenland, Neuhaus/Klausen	MN812828.1	COI_2	MN812833.1	Cytb_1	present study
Austria, Vienna	MN812829.1	COI_1	MN812834.1	Cytb_1	present study
Austria, Styria, Laafeld	MN812830.1	COI_2	MN812835.1	Cytb_1	present study
Austria, Styria, Hummersdorf	MT332686.1	COI_1	MT332689.1	Cytb_1	present study
Austria, Styria, Bad Radkersburg	MT332687.1	COI_1	MT332690.1	Cytb_1	present study
Austria, Styria, Unterpurkla	MT332688.1	COI_2	MT332691.1	Cytb_1	present study

## Supplementary Materials

The following are available online at https://www.mdpi.com/2076-0817/9/12/1032/s1, Table S1. Surveyed locations in Austria. Table S2. Included *COI* sequences of *Transphlebotomus* for inter- and intraspecific distance calculations. Table S3. Included *cyt b* sequences of *Transphlebotomus* for inter- and intraspecific distance calculations. Table S4. Included sand fly specimens to MALDI-TOF mass spectrometry.

## Abbreviations

*COI*: cytochrome c oxidase subunit I, *cytb*: cytochrome b, Hd: haplotype diversity, MALDI-TOF MS: matrix-assisted laser desorption/ionization time of flight mass spectrometry, RH: relative humidity, SD: standard deviation.
