# Supplementary material for: Integrative Approach to Phlebotomus mascittii Grassi, 1908: First Record in Vienna with New Morphological and Molecular Insights
Source: Pathogens. 2020 Dec 9;9(12):1032. doi: 10.3390/pathogens9121032 (PMC7764109; doi:10.3390/pathogens9121032)
Supplement: Supplementary file 1 [file pathogens-09-01032-s001.zip › Supplementary figure captions.docx]

**Supplementary file captions**

**Supplementary file 1.** Results of PCR targeting the *COI* gene of various *Ph. mascittii* specimens analyzed in this study. (M) 50–3000 bp step marker, (1–12) *Ph. mascittii* samples from Austria, (N) negative control.

**Supplementary file 2.** Results of PCR targeting the *Cytb* gene of various *Ph. mascittii* specimens analyzed in this study. (M) 50–3000 bp step marker, (1–12) *Ph. mascittii* samples from Austria, (N) negative control.
