# Supplementary material for: Integrative Approach to Phlebotomus mascittii Grassi, 1908: First Record in Vienna with New Morphological and Molecular Insights
Source: Pathogens. 2020 Dec 9;9(12):1032. doi: 10.3390/pathogens9121032 (PMC7764109; doi:10.3390/pathogens9121032)
Supplement: Supplementary file 1 [file pathogens-09-01032-s001.zip › Supplementary tables captions.docx]

**Supplementary file captions**

**Table S1.** Surveyed locations in Austria.

**Table S2.** Included *COI* sequences of *Transphlebotomus* for inter- and intraspecific distance calculations.

**Table S3.** Included *cyt b* sequences of *Transphlebotomus* for inter- and intraspecific distance calculations.

**Table S4.** Included sand fly specimens to MALDI-TOF mass spectrometry.
