# Supplementary figures and images for: Integrative Approach to Phlebotomus mascittii Grassi, 1908: First Record in Vienna with New Morphological and Molecular Insights
Source: Pathogens. 2020 Dec 9;9(12):1032. doi: 10.3390/pathogens9121032 (PMC7764109; doi:10.3390/pathogens9121032)

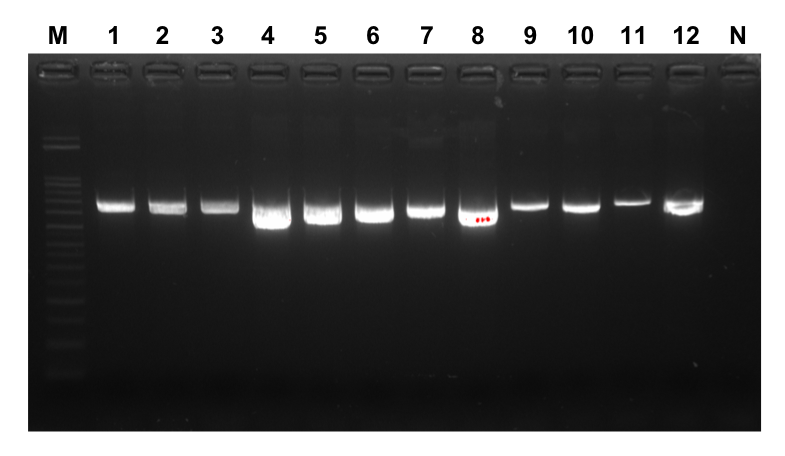

Supplement: Supplementary file 1 [file pathogens-09-01032-s001.zip › Suppl Fig 1. COI PCR Gel.png]

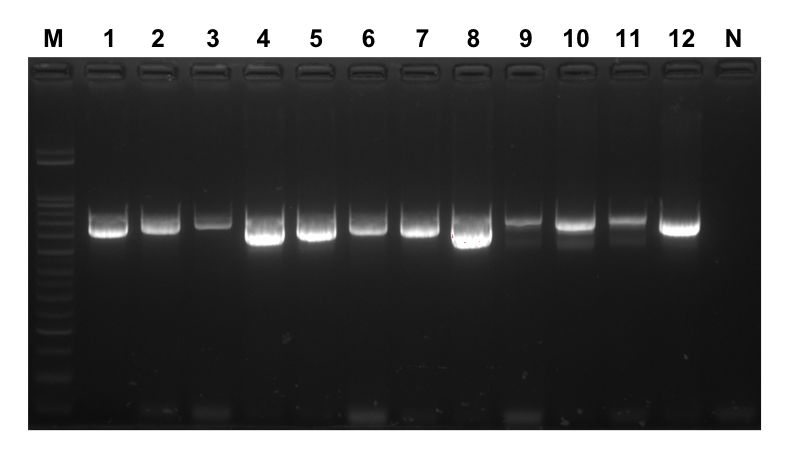

Supplement: Supplementary file 1 [file pathogens-09-01032-s001.zip › Suppl Fig 2. Cytb PCR Gel.png]
